# Role of nerve growth factor on cognitive impairment in patients with Alzheimer's disease carrying apolipoprotein E ε4

**DOI:** 10.1111/cns.14560

**Published:** 2023-12-19

**Authors:** Mingyue He, Zhan Liu, Tenghong Lian, Peng Guo, Wenjing Zhang, Yue Huang, Yanan Zhang, Gaifen Liu, Weijiao Zhang, Jinghui Li, Huiying Guan, Weijia Zhang, Dongmei Luo, Jing Qi, Hao Yue, Xiaomin Wang, Wei Zhang

**Affiliations:** ^1^ Department of Neurology Beijing Tiantan Hospital, Capital Medical University Beijing China; ^2^ Center for Cognitive Neurology, Department of Neurology Beijing Tiantan Hospital, Capital Medical University Beijing China; ^3^ China National Clinical Research Center for Neurological Diseases Beijing Tiantan Hospital, Capital Medical University Beijing China; ^4^ Department of Pharmacology, School of Medical Sciences, Faculty of Medicine & Health UNSW Sydney Sydney New South Wales Australia; ^5^ Department of Blood Transfusion Beijing Tiantan Hospital, Capital Medical University Beijing China; ^6^ Department of Physiology Capital Medical University Beijing China; ^7^ Center of Parkinson's Disease Beijing Institute for Brain Disorders Beijing China; ^8^ Beijing Key Laboratory on Parkinson Disease Beijing China

**Keywords:** Alzheimer's disease, *apolipoprotein E* ε4, cognitive function, nerve growth factor, neurotrophic factors

## Abstract

**Aims:**

To investigate the roles of neurotrophic factors on cognition in patients with Alzheimer's disease (AD) carrying *Apolipoprotein E (APOE)* ε4.

**Methods:**

Totals of 173 patients with AD were divided into *APOE* ε4 carrier and non‐carrier groups, and their demographics, cognition, and neurotrophic factors in cerebrospinal fluid (CSF) were compared. Multiple linear regression analyses were performed to assess correlations among *APOE* ε4, neurotrophic factors and cognition. Mediation analyses were conducted to assess the sequential associations among *APOE* ε4, nerve growth factor (NGF), and cognition.

**Results:**

Global cognition and multiple domains were impaired in the *APOE* ε4 carrier group (all *p* < 0.05). NGF level in the *APOE* ε4 carrier group was lower than that in the non‐carrier group (*p* = 0.016). NGF level showed significant correlations with both global and multiple domains cognitions. Specifically, NGF mediated the association between *APOE* ε4 and Animal Fluency Test score (*β*, −0.45; 95% CI [−0.96, −0.07]; *p* < 0.001) and Trail Making Test‐A (time) (*β*, 0.15; 95% CI [0.01, 0.33]; *p* < 0.001).

**Conclusion:**

*APOE* ε4 is associated with cognitive impairment, and those carrying *APOE* ε4 have decreased NGF level in CSF. Declined NGF level is correlated with compromised cognition. NGF mediates *APOE* ε4‐associated cognitive impairment.

## Graphical abstract

*Apolipoprotein E (APOE)* ε4 was associated with cognitive impairment. Patients with Alzheimer's disease carrying *APOE* ε4 had decreased nerve growth factor (NGF) levels in cerebrospinal fluid. Declined NGF is correlated with compromised cognitive function, including global cognition and multiple cognitive domains. NGF mediated *APOE* ε4‐associated cognitive impairment.

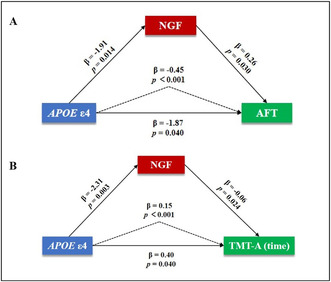

## 1 INTRODUCTION

Alzheimer's disease (AD) is the most common cognitive disorder among the elderly. Typical patients with AD are initially characterized by episodic memory decline, followed by overall cognitive impairment, neuropsychiatric symptoms, and impaired activities of daily living.^1^

*Apolipoprotein E (APOE)*, encoding the indispensable lipid transporter APOE protein in the brain, includes three alleles of ε2, ε3, and ε4, of which, *APOE* ε4 is the strongest risk gene for developing sporadic AD.^2^ *APOE* ε4 carriers exhibit an earlier age of onset, a faster rate of cognitive decline, and a poorer cognitive outcome.^2^, ^3^ Previous studies have linked *APOE* ε4 to synaptic dysfunction and aggravated cognitive impairment by promoting depositions of neuropathological proteins of AD^4^, ^5^ and eliciting neuroinflammation.^6^ However, the role of *APOE* ε4 on the neurotrophic state in the brain remains unclear.

Neurotrophic factors constitute a family of proteins, including brain‐derived neurotrophic factor (BDNF), glial cell‐derived neurotrophic factor (GDNF), and nerve growth factor (NGF). These factors played crucial roles in the survival, growth, and differentiation of neurons during development.^7^ Decreased levels of BDNF and its precursor were observed in several brain regions and peripheral blood of patients with AD.^8^ GDNF level was elevated in the cerebrospinal fluid (CSF) of early‐stage patients with AD but decreased in the middle temporal gyrus and serum of patients with AD.^9^ In addition, proNGF levels in the cerebral cortex and CSF of patients with AD were increased, while NGF mRNA was unchanged in the cerebral cortex of patients with AD.^10^, ^11^, ^12^ It came to the limelight that abnormal secretions of these neurotrophic factors were correlated with the progression of neuropathology and cognitive impairment of patients with AD.^13^, ^14^ However, the relationships between neurotrophic factors in CSF, *APOE* ε4, and functions of different cognitive domains remain unknown.

In this study, we hypothesized that both *APOE* ε4 and neurotrophic factors might be related to cognitive impairment, *APOE* ε4 might be associated with decreased neurotrophic factors in CSF, and the declined neurotrophic factors might play a pivotal role in mediating *APOE* ε4‐associated cognitive impairment. Based on this hypothesis, patients with AD were divided into *APOE* ε4 carrier and *APOE* ε4 non‐carrier groups. Demographic variables were collected, overall cognitive function and individual cognitive domains were assessed by a series of rating scales, and the levels of neurotrophic factors, including BDNF, GDNF, and NGF in CSF were measured by enzyme‐linked immunosorbent assay (ELISA) with aims to figure out the roles of neurotrophic factors on cognitive impairment in patients with AD carrying *APOE* ε4.

## 2 METHODS

### 2.1 Ethics statement

The study was approved by the Ethical Review Board of Beijing Tiantan Hospital. Written informed consent was obtained from patients and their caregivers. All the procedures were conducted in accordance with the guidelines and regulations of ethical principles for medical research involving human subjects of the Declaration of Helsinki.

### 2.2 Participants

Patients diagnosed with AD according to the National Institute of Aging and Alzheimer's Association (NIA‐AA) criteria^15^, ^16^ were consecutively enrolled from the Center for Cognitive Neurology, Department of Neurology, Beijing Tiantan Hospital, Capital Medical University. The exclusion criteria included: (1) Patients with a history of diseases that might affect cognitive function besides AD, such as cerebrovascular diseases, Lewy body diseases, frontotemporal neurodegeneration, corticobasal degeneration, multiple sclerosis, epilepsy, substance abuse, etc. (2) Patients with severe systematic diseases, active infections, chronic wasting disease, autoimmune diseases, hematological system diseases, and malignant tumor. (3) Patients suffered from traumatic brain injury and major surgery recently. (4) Patients were undergoing steroid treatment. (5) Patients were unable to cooperate with all the examinations for various reasons.

### 2.3 Collection of demographic variables

Demographic variables, including gender, age, age of onset, disease duration, education level, drinking, smoking, and body mass index (BMI), were collected. In addition, a history of hypertension, hyperlipidemia, myocardial infarction, atrial fibrillation, diabetes mellitus, et al., was also collected.

### 2.4 Assessment of cognitive function

The overall cognitive function of patients with AD was assessed by the Mini‐Mental State Examination (MMSE)^17^ and the Montreal Cognitive Assessment (MoCA).^18^ In the individual cognitive domains, verbal memory was evaluated by the Auditory Verbal Learning Test (AVLT), and visual delayed memory was evaluated by the Rey‐Osterreithm Complex Figure Test (RCFT)‐delayed recall.^19^, ^20^ The language was evaluated by the Animal Fluency Test (AFT), the Verbal Fluency Test (VFT), and the Boston Naming Test (BNT).^21^, ^22^ Attention was evaluated by the Symbol Digit Modalities Test (SDMT),^23^ the Trail Making Test (TMT)‐A,^24^ as well as the Stroop Color‐Word Test (SCWT)‐A and the SCWT‐B.^25^ Visuospatial ability was evaluated by the RFT. Executive function was evaluated by the SCWT‐C and the TMT‐B. A detailed description of these rating scales was provided in the Supplementary material.

### 2.5 Detections of *APOE* genotypes

The venous blood samples of patients with AD were collected from the median elbow under fasting conditions in the morning following admission and then sent to the clinical laboratory of our hospital.

Genotyping for *APOE* single nucleotide variants (rs429358 C/T and rs7412 C/T), which define *APOE* 𝜀2, 𝜀3, and 𝜀4, was performed by real‐time fluorescence quantitative polymerase chain reaction using nucleic acid detection reagents (Youzhiyou company, Wuhan, China).^26^

### 2.6 Collections of CSF samples

Patients were requested to withdraw anti‐cognitive impairment drugs for at least 12–14 hours before lumbar puncture if their conditions allowed. CSF samples were collected under fasting condition through lumbar puncture, followed by being immediately centrifuged at 4°C with 1500 *g* for 10 min. Each CSF sample was then allocated into separate Nunc cryotubes (Beijing JingkeHongda Biotechnology Co., Ltd, Beijing, China) and frozen for 0.5 mL per tube at −80°C until the assay.^27^

### 2.7 Measurements of neurotrophic factors in CSF

Neurotrophic factors, including BDNF (ProcartaPlex™ Multiplex Immunoassay Kit, Invitrogen, USA), GDNF (ProcartaPlex™ Multiplex Immunoassay Kit, Invitrogen, USA), and NGF (Human NGF DuoSet ELISA Kit, R&D Systems, USA), in CSF were measured by ELISA.

### 2.8 Statistical analysis

Statistical analyses were performed by SPSS Statistics 25.0 (IBM Corporation, New York, USA). Statistical significance was defined as a two‐sided *p* < 0.05.

The data were tested for normal distribution using the Kolmogorov–Smirnov test. Demographic variables, cognitive function, and neurotrophic factors in CSF were compared between *APOE* ε4 carrier and non‐carrier groups. Continuous variables conforming to normal distribution were presented as means ± standard deviations (SD) and compared by two‐tailed *t‐*test, while non‐normal distributed variables were presented as median (quartile) and compared by a non‐parametric test, and categorical variables were presented as number (percentage) and compared by Chi‐Squared test. Multiple linear regression analyses were performed to assess the correlations among *APOE* ε4, neurotrophic factors in CSF, and cognitive function of patients with AD. Mediation analysis was conducted to evaluate the sequential associations among *APOE* ε4, NGF, and cognitive function.

## 3 RESULTS

### 3.1 Demographic variables in patients with AD

A total of 173 patients with AD were enrolled in this study, of whom 55 cases (31.79%) carried *APOE* ε4, 34 cases (61.83%) were female, the mean age was 65.49 ± 9.00 years old, and the median disease duration was 24.00 (12.00, 48.00) months in *APOE* ε4 carrier group. Demographic variables, including gender, age, age of onset, disease duration, education level, smoking, drinking, BMI, etc., were not significantly different between the two groups (Table 1).

**TABLE 1:** Demographic variables of *APOE* ε4− and *APOE* ε4+ groups.

	*APOE* ε4− group (*n* = 118)	*APOE* ε4+ group (*n* = 55)	*p*
Female (*n* [%])	68.00 (57.63)	34.00 (61.82)	0.602
Age (years, mean ± SD)	62.71 ± 9.05	65.49 ± 9.00	0.062
Age of onset (years, median [quartile])	59.00 (53.00, 64.75)	64.00 (53.50, 70.00)	0.075
Disease duration (months, median [quartile])	25.00 (13.50, 49.00)	24.00 (12.00, 48.00)	0.460
Education level			0.311
Primary school and below (*n* [%])	25 (21.19)	11 (20.00)	
Middle and high school (*n* [%])	55 (46.61)	21 (38.18)	
Bachelor's degree and above (*n* [%])	38 (32.20)	13 (23.64)	
Smoking (*n* [%])	31 (26.27)	15 (27.27)	0.853
Drinking (*n* [%])	28 (23.73)	14 (25.45)	0.832
BMI (median [quartile])	23.89 (21.91, 25.87)	23.10 (21.60, 25.16)	0.252
History			
Hypertension (*n* [%])	39 (33.05)	13 (23.63)	0.215
Hyperlipidemia (*n* [%])	15 (12.71)	13 (23.63)	0.142
Myocardial infarction (*n* [%])	2 (1.69)	0 (0.00)	1.000
Atrial fibrillation (*n* [%])	0 (0.00)	1 (1.82)	0.308
Diabetes mellitus (*n* [%])	15 (12.71)	6 (10.91)	0.769
Hyperhomocysteinemia (*n* [%])	3 (2.54)	0 (0.00)	0.553
Cerebrovascular disease (*n* [%])	17 (14.41)	5 (9.10)	0.357
Thyroid disease (*n* [%])	8 (6.78)	4 (7.27)	0.968
Chronic obstructive pulmonary disease (*n* [%])	2 (1.69)	0 (0.00)	1.000
Asthma (*n* [%])	3 (2.54)	2 (3.64)	0.411
Insomnia (*n* [%])	16 (13.56)	3 (5.45)	0.074
Sleep apnea syndrome (*n* [%])	4 (4.24)	1 (1.82)	0.588
Depression (*n* [%])	8 (6.78)	3 (5.45)	0.564
Other mental disorders (*n* [%])	3 (2.54)	0 (0.00)	0.223

*Note*: Data were presented as number (percentage), means ± SD, or median (quartile). *APOE* ε4−, *APOE* ε4 non‐carriers; *APOE* ε4+, *APOE* ε4 carriers.

Abbreviations: *APOE*, apolipoprotein E; BMI, body mass index; SD, standard deviation.

### 3.2 Association of *APOE* ε4 with cognitive function

A comparison of cognitive function between *APOE* ε4 carrier and *APOE* ε4 non‐carrier groups was presented (Table 2). Regarding global cognitive function, MMSE (*p* = 0.042) and MoCA scores (*p* = 0.015) in *APOE* ε4 carrier group were lower than those in the *APOE* ε4 non‐carrier group. In terms of individual cognitive domain function, compared with *APOE* ε4 non‐carrier group, *APOE* ε4 carrier group had the lower scores of AVLT N1‐3 (*p* = 0.012), AVLT N4 (*p* = 0.027), AVLT N1‐5 (*p* = 0.003) and AVLT N6 (*p* = 0.013), lower scores of AFT (*p* = 0.011), VFT‐H (*p* = 0.044) and VFT‐alternating fluency (*p* = 0.046), and longer time for TMT‐A (*p* = 0.034) and TMT‐B (*p* = 0.023).

**TABLE 2:** Cognitive function of *APOE* ε4− and *APOE* ε4+ groups.

	*APOE* ε4− group (*n* = 118)	*APOE* ε4+ group (*n* = 55)	*p*
Global cognitive function			
MMSE (points, median [quartile])	20.50 (13.00, 25.00)	18.00 (10.00, 22.00)	0.042*
MoCA (points, mean ± SD)	14.44 ± 6.53	11.69 ± 6.32	0.015*
Individual cognitive domain function			
Memory			
AVLT N1‐3 (points, mean ± SD)	10.55 ± 5.84	8.86 ± 5.93	0.012*
AVLT N4 (points, median [quartile])	0.00 (0.00, 3.00)	0.00 (0.00, 1.00)	0.027*
AVLT N5 (points, median [quartile])	0.00 (0.00, 4.00)	0.00 (0.00, 1.00)	0.073
AVLT N1‐5 (points, median [quartile])	12.00 (8.00, 20.00)	8.50 (3.00, 14.25)	0.003**
AVLT N6 (points, median [quartile])	0.00 (0.00, 3.00)	0.00 (0.00, 0.75)	0.013*
AVLT N7 (points, median [quartile])	9.00 (6.00, 12.00)	8.00 (0.25, 10.75)	0.074
RCFT‐delayed (points, median [quartile])	0.00 (0.00, 10.00)	0.00 (0.00, 5.00)	0.467
Language			
AFT (points, mean ± SD)	11.79 ± 5.81	9.73 ± 6.10	0.011*
VFT‐H (points, median [quartile])	9.00 (6.00, 15.00)	8.50 (5.00, 12.00)	0.044*
VFT‐alternating fluency (points, mean ± SD)	7.59 ± 5.34	6.38 ± 5.15	0.046*
BNT (points, median [quartile])	21.00 (18.00, 26.00)	21.00 (15.00, 24.00)	0.209
Visuospatial ability			
RCFT‐imitation (points, median [quartile])	26.00 (1.00, 33.00)	20.25 (0.00, 32.25)	0.622
Attention/Executive function			
TMT‐A (points, median [quartile])	25.00 (22.00, 25.00)	25.00 (20.25, 25.00)	0.683
TMT‐A (time) (minutes, median [quartile])	1.88 (1.05, 3.70)	2.55 (1.45, 4.00)	0.034*
TMT‐B (points, median [quartile])	21.00 (5.50, 25.00)	15.00 (6.00, 24.00)	0.184
TMT‐B (time) (minutes, median [quartile])	2.99 (1.18, 4.00)	4.00 (2.90, 4.00)	0.023*
SCWT‐A (points, median [quartile])	50.00 (49.00, 50.00)	50.00 (48.00, 50.00)	0.262
SCWT‐A (time) (minutes, median [quartile])	0.67 (0.48, 0.94)	0.79 (0.50, 1.06)	0.263
SCWT‐B (points, median [quartile])	50.00 (47.25, 50.00)	50.00 (46.00, 50.00)	0.523
SCWT‐B (time) (minutes, median [quartile])	0.88 (0.62, 1.20)	0.93 (0.74, 1.51)	0.461
SCWT‐C (points, median [quartile])	47.00 (37.00, 49.00)	43.00 (26.00, 47.50)	0.177
SCWT‐C (time) (minutes, median [quartile])	1.55 (0.91, 2.11)	1.74 (0.98, 2.52)	0.515
SDMT (points, median [quartile])	20.00 (0.75, 31.75)	15.00 (0.00, 26.50)	0.318

*Note*: Data were presented as means ± SD or median (quartile). APOE ε4−, APOE ε4 non‐carriers; APOE ε4+, APOE ε4 carriers.

Abbreviations: AFT, Auditory Verbal Learning Test; *APOE*, apolipoprotein E; AVLT, Animal Fluency Test; BNT, Boston Naming Test; MMSE, Mini‐Mental State Examination; MoCA, Montreal Cognitive Assessment; RCFT, Rey‐Osterrieth Complex Figure Test; SCWT, Stroop Color and Word Test; SD, standard deviation; SDMT, Symbol Digit Modalities Test; TMT, Trail Making Test; VFT‐H, Verbal Fluency Test‐Household.

*p < 0.05, **p < 0.01.

Further multiple linear regression analyses revealed the association between *APOE* ε4 and cognitive function after adjusting for age, gender, age of onset, disease duration, and education level (Table S1). In global cognitive function, *APOE* ε4 was associated with a lower MoCA score (*β*, −2.31; 95% CI [−4.58, −0.05]; *p* = 0.046). In individual cognitive domains, *APOE* ε4 was associated with the lower scores of AVLT N1‐3 (*β*, −2.35; 95% CI [−4.51, −0.18]; *p* = 0.034), AVLT N1‐5 (*β*, −4.20; 95% CI [−8.14, −0.25]; *p* = 0.037), AVLT N6 (*β*, −1.01; 95% CI [−1.94, −0.08]; *p* = 0.034) and AVLT N7 (*β*, −2.62; 95% CI [5.02, −0.21]; *p* = 0.033), the lower score of AFT (*β*, −2.06; 95% CI [−4.04, −0.09]; *p* = 0.040), and longer time spend on TMT‐A (*β*, 0.45; 95% CI [0.21, 0.88]; *p* = 0.040). *APOE* ε4 showed no significant association with the scores of rating scales for language and visuospatial ability.

### 3.3 Association of *APOE* ε4 with the levels of neurotrophic factors in CSF

**FIGURE 1:**
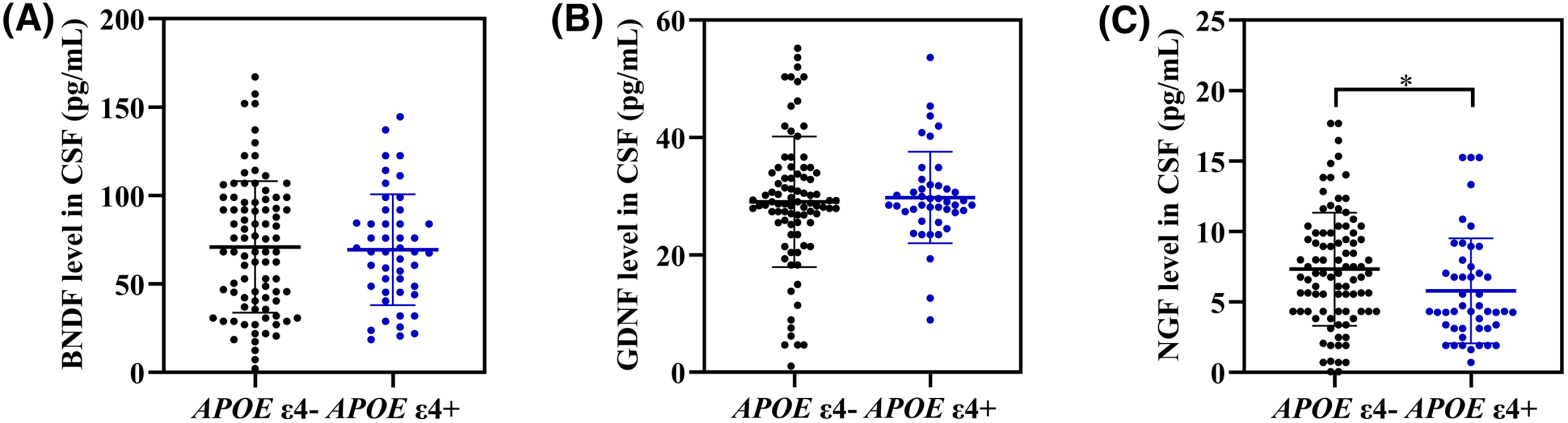
The levels of neurotrophic factors in CSF from *APOE* ε4− and *APOE* ε4+ groups. Comparison of the levels of BDNF (A), GDNF (B), and NGF (C) in CSF between *APOE* ε4− and *APOE* ε4+ groups. *APOE* ε4−, *APOE* ε4 non‐carriers; *APOE* ε4+, *APOE* ε4 carriers. *APOE*, apolipoprotein E; BDNF, brain‐derived neurotrophic factor; CSF, cerebrospinal fluid; GDNF, glial cell‐derived neurotrophic factor; NGF, nerve growth factor. *p < 0.05.

The levels of neurotrophic factors in CSF were compared between *APOE* ε4 carrier and *APOE* ε4 non‐carrier groups. NGF level in *APOE* ε4 carrier group was significantly lower than that in *APOE* ε4 non‐carrier group (*p* = 0.016) (Figure 1).

AD patients were further divided into *APOE* ε4−/−, *APOE* ε4+/− and *APOE* ε4+/+ groups. It was found that the NGF level in *APOE* ε4+/− group was significantly lower than that in *APOE* ε4−/− group (*p* = 0.042). Moreover, *APOE* ε4+/+ group demonstrated the lowest NGF level in CSF compared to the other groups (Figure S1).

Multiple linear regression analyses illustrated that *APOE* ε4 was associated with a declined NGF level in CSF after adjusting for age, gender, age of onset, disease duration, education level, and BMI (*β*, −1.98; 95% CI [−3.78, −0.19]; *p* = 0.031) (Table 3).

**TABLE 3:** Association between *APOE* ε4 and neurotrophic factors in CSF in patients with AD.

	Unadjusted		Adjusted	
	*β* (95% CI)	*p*	*β* (95% CI)	*p*
BDNF (pg/ml)	−0.09 (−14.60, 14.43)	0.991	−7.25 (−24.56, 10.05)	0.408
GDNF (pg/ml)	2.28 (−2.65, 7.21)	0.363	3.38 (−3.36, 10.12)	0.322
NGF (pg/ml)	−1.87 (−3.39, −0.35)	0.016*	−1.98 (−3.78, −0.19)	0.031*

*Note*: Age, gender, age of onset, disease duration, education level, and BMI were adjusted.

Abbreviations: *APOE*, apolipoprotein E; AD, Alzheimer's disease; BDNF, brain‐derived neurotrophic factor; CI, confidence interval; CSF, cerebrospinal fluid; GDNF, glial cell‐derived neurotrophic factor; NGF, nerve growth factor.

* *p* < 0.05.

### 3.4 Association between NGF level in CSF and cognitive function

**FIGURE 2:**
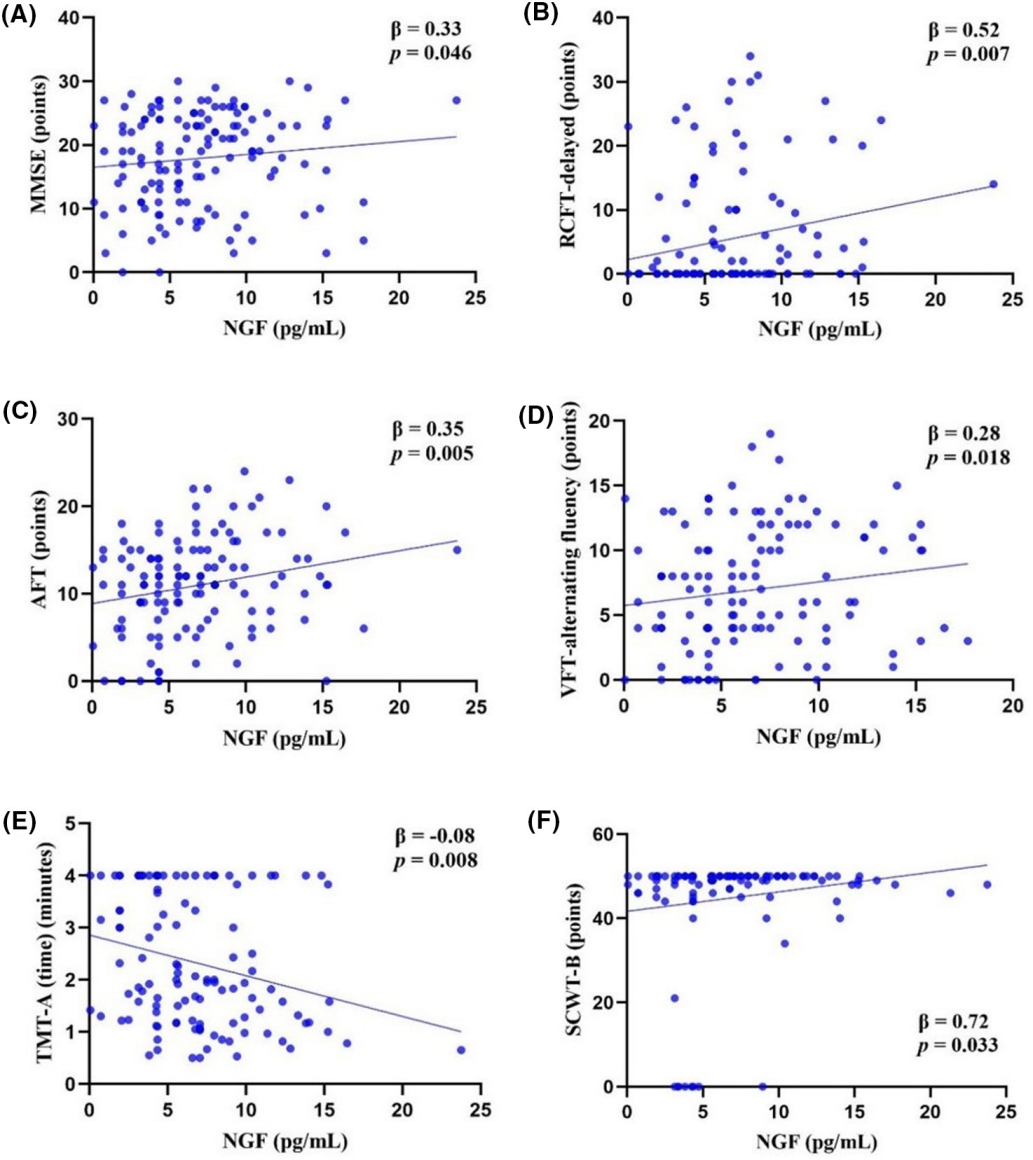
Association between NGF level in CSF and cognitive function in patients with AD. Multiple linear regression analyses were performed between NGF level and MMSE (A), RCFT‐delayed (B), AFT (C), VFT‐alternating fluency (D), TMT‐A (time) (E), and SCWT‐B (F) in patients with AD after adjusting for age, gender, age of onset, disease duration, education level, and BMI. AD, Alzheimer’s disease; AFT, Animal Fluency Test; CSF, cerebrospinal fluid; MMSE, Mini‐Mental State Examination; NGF, nerve growth factor; RCFT, Rey‐Osterrieth Complex Figure Test; SCWT, Stroop Color and Word Test; TMT, Trail Making Test; VFT, Verbal Fluency Test.

Multiple linear regression analyses were conducted to explore the association between NGF level in CSF and cognitive function in patients with AD after adjusting for age, gender, age of onset, disease duration, education level, and BMI (Figure 2). Regarding global cognitive function, NGF level was positively associated with MMSE score (*β*, 0.33; 95% CI [0.01, 0.66]; *p* = 0.046). As far as individual cognitive domain, NGF level was positively associated with the scores of RCFT‐delayed (*β*, 0.52; 95% CI [0.15, 0.89]; *p* = 0.007), AFT (*β*, 0.35; 95% CI [0.107, 0.585]; *p* = 0.005) and VFT‐alternating fluency (*β*, 0.28; 95% CI [0.05, 0.52]; *p* = 0.018), which reflected language function. Furthermore, NGF level was negatively associated with TMT‐A (time) (*β*, −0.08; 95% CI [−0.14, −0.02]; *p* = 0.008) and positively associated with SCWT‐B score (*β*, 0.72; 95% CI [0.06, 1.37]; *p* = 0.033), which reflected attention/executive function.

Within the subgroup with patients carrying *APOE* ε4, NGF level was positively associated with the scores of AVLT N1‐3 (*β*, 0.50; 95% CI [0.11, 0.89]; *p* = 0.015), AVLT N4 (*β*, 0.20; 95% CI [0.05, 0.34]; *p* = 0.010), AVLT N1‐5 (*β*, 0.63; 95% CI [0.03, 1.24]; *p* = 0.042), RCFT‐delayed (*β*, 1.29; 95% CI [0.37, 2.22]; *p* = 0.010), and VFT‐alternating fluency (*β*, 0.41; 95% CI [0.02, 0.79]; *p* = 0.041). NGF level was not associated with visuospatial ability and attention/executive function (Table S2).

### 3.5 Mediation analyses among *APOE* ε4, NGF level in CSF, and cognitive function

**FIGURE 3:**
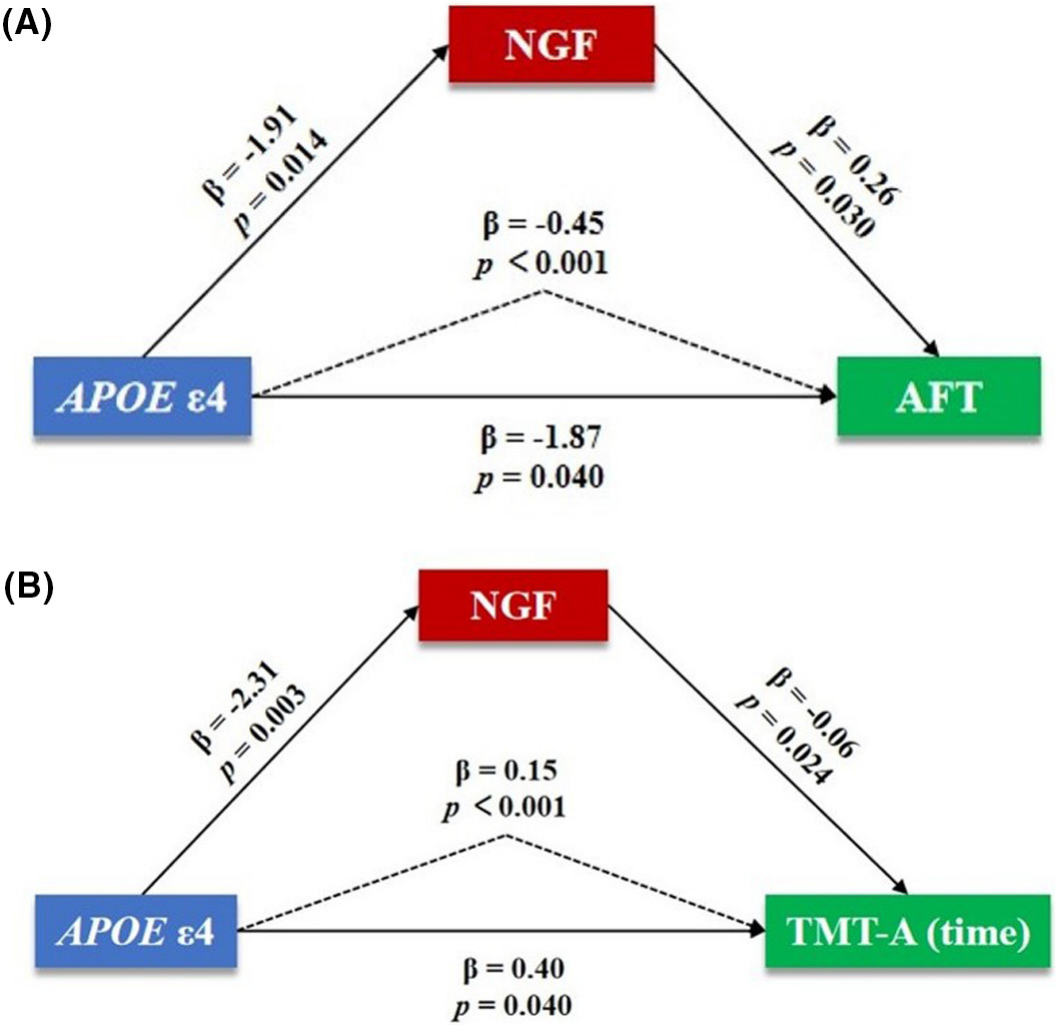
Association among *APOE* ε4, NGF level in CSF and cognitive function in patients with AD. Mediation analyses among *APOE* ε4, NGF level in CSF, and AFT score (A) and TMT‐A (time) (B) in patients with AD after adjusting for age, gender, age of onset, disease duration, education level, and BMI. AFT, Animal Fluency Test; AD, Alzheimer's disease; *APOE*, apolipoprotein E; CSF, cerebrospinal fluid; NGF, nerve growth factor; TMT, Trail Making Test.

Mediation analyses were conducted to assess the sequential associations among *APOE* ε4, NGF level in CSF, and cognitive function in patients with AD (Figure 3). The results showed that NGF level in CSF significantly mediated the associations between *APOE* ε4 and AFT (*β*, −0.45; 95% CI [−0.96, −0.07]; *p* < 0.001) (Figure 3A) as well as TMT‐A (time) (*β*, 0.15; 95% CI [0.01, 0.33]; *p* < 0.001) (Figure 3B) after adjusting for age, gender, age of onset, disease duration, education level and BMI. There was no notable mediation effect of NGF level in CSF on the associations between *APOE* ε4 and functions of other cognitive domains.

## 4 DISCUSSION

In this study, our exploration delved into the association among *APOE* ε4, the levels of neurotrophic factors in CSF, and cognitive function in patients with AD. The findings revealed that: (1) *APOE* ε4 was significantly associated with poorer cognitive function; (2) *APOE* ε4 was associated with a declined NGF level in CSF; (3) The declined NGF level was significantly associated with cognitive impairment; (4) The decline of NGF level mediated significant association between *APOE* ε4 and impairments of multiple cognitive domains.

### 4.1 Relationship between *APOE* ε4 and cognitive impairment in patients with AD

It is well known that *APOE* ε4 is the strongest risk gene for sporadic AD.^2^ Previous studies demonstrated that patients with AD with *APOE* ε4 had poorer cognitive function.^28^, ^29^, ^30^ Nonetheless, the precise relationships between *APOE* ε4 and functions of cognitive domains remained elusive. In this study, we comprehensively evaluated individual cognitive domains by a body of rating scales and found that patients with AD carrying *APOE* ε4 had particularly more severe memory impairment, which was potentially attributed to the close association of *APOE* ε4 with temporal lobe (especially hippocampus) atrophy and dysfunction of default mode network.^28^, ^29^, ^30^ In addition to memory, our novel observation indicated a significant association between *APOE* ε4 and impaired cognitive domains related to language and attention/executive function. These findings might be due to the faster rate of cognitive decline and poorer cognitive function that *APOE* ε4 carriers had.^2^, ^3^

### 4.2 Relationship between *APOE* ε4 and the levels of neurotrophic factors in CSF in patients with AD

Within the scope of various neurotrophic factors measured in this study, NGF level in CSF from patients with AD with *APOE* ε4 was significantly declined compared to those without *APOE* ε4. APOE, a pivotal lipid transport protein in the brain, is secreted primarily by astrocytes and to a lesser extent by microglia. APOE binds with two major metabolic receptors, the low‐density lipoprotein receptor and lipoprotein receptor‐related protein, facilitating lipid transport between cells, redistributing intracellular lipids, and maintaining lipid balance in the brain.^31^ Compared to other APOE isoforms, APOE4 encoded by *APOE* ε4 exerted the weakest effect on lipid transport.^32^ Cholesterol homeostasis is essential for the brain, with insufficient accumulation leading to abnormal cellular nutrition and energy metabolism, and excessive accumulation resulting in synaptic plasticity damage and neuronal apoptosis.^33^ However, limited studies directly address the relationship between *APOE* ε4 or APOE4 and NGF. Based on the results of this investigation, we speculate that the impaired cellular nutrient and energy metabolism, stemming from the weakened lipid transport capacity of APOE4, may contribute to the decrease of NGF level in CSF of patients with AD.

### 4.3 Relationship between NGF level in CSF and cognitive impairment in patients with AD

In this study, we found a close relationship between NGF level in CSF and the cognitive function of patients with AD. It is well known that the main source of acetylcholine in hippocampus and cerebral cortex is the forebrain cholinergic neurons, which dysfunction or degeneration is the prominent cause of decreased acetylcholine level and impaired cognitive function in patients with AD.^1^ NGF, a member of the neurotrophin family, supplies the forebrain cholinergic neurons to maintain their cholinergic phenotype. Degeneration of the cholinergic system is associated with dysregulation of the NGF metabolic pathway, which controls the maturation and degradation of NGF.^34^ Previous studies have indicated that the disturbed regulation of NGF pathway, including the impaired maturation and increased degradation of NGF, existed in AD, even in the preclinical stage of the disease.^35^, ^36^ NGF deprivation was associated with poorer cognitive function, and treatment with NGF ameliorated neuropathology and thereafter inhibited memory decline in AD animal models.^11^, ^37^ However, the relationships between NGF and functions of different cognitive domains remain unclear. In this study, we observed, for the first time, a strong association between NGF level in CSF and impaired global cognition and functions of multiple cognitive domains, including memory, language, attention, and executive function. In addition, the declined NGF level was particularly and fairly associated with compromised memory and language in patients with AD carrying *APOE* ε4. At the early stage of AD, cognitive domains, such as memory and language, are specifically vulnerable to the decrease of NGF in the individuals carrying *APOE* ε4, as indicated by the results from this study. The detailed mechanisms underlying their association warrant further investigation in the future.

### 4.4 Role of NGF on *APOE* ε4‐associated cognitive impairment in patients with AD

Previous studies highlighted that *APOE* ε4 exacerbated cognitive impairment by promoting the depositions of neuropathological proteins in AD and facilitating neuroinflammation by activating microglia and astrocytes.^4^, ^5^, ^6^ However, the roles of neurotrophic factors on *APOE* ε4 and the associated cognitive impairment remain unclear. In the current study, we for the first time found that the declined NGF level in CSF played an important role in mediating *APOE* ε4‐associated impairments of overall cognition and function of cognitive domains, particularly language and attention/executive function. Given that APOE4 has poorer lipid transport capacity than other APOE isoforms, leading to lipid instability in cells, we speculate that APOE4 may be responsible for the declined NGF level in the brain, and NGF deficiency may further accelerate degeneration of the cholinergic system, eventually propagating cognitive impairment in patients with AD. The potential mechanisms illustrating the mediating role of NGF on *APOE* ε4‐associated impairments of language and executive function remain to be elucidated.

### 4.5 Limitations

This study had limitations. First, while measuring neurotrophic factors in CSF is one of the most objective ways to capture their changes in the brain, it is tough to obtain CSF from elderly patients with AD, particularly from people with normal cognition. We will focus on collecting more CSF samples from a larger cohort of patients with AD and cognitively normal controls. Second, unlike a randomized controlled design, this cross‐sectional study may introduce relative selection bias.

## 5 CONCLUSION

The results from this study indicate a significant association between *APOE* ε4 and compromised cognitive function in patients with AD. Patients with AD carrying *APOE* ε4 have prominently decreased NGF level in CSF. Decreased NGF level is markedly associated with drastically impaired overall cognition and individual cognitive domains, including memory, language, and attention/executive function. More importantly, NGF plays a pivotal role in mediating remarkably compromised cognitive function associated with *APOE* ε4. These novel findings contribute to a better understanding of the mediating role of NGF deficiency on cognitive impairment of patients with AD carrying *APOE* ε4, thus, replenishing NGF may be an effective therapy for slowing down cognitive impairment in patients with AD carrying *APOE* ε4.

## AUTHOR CONTRIBUTIONS

Mingyue He and Zhan Liu contributed to the conception, design, and data statistics of the study and paper writing; Tenghong Lian, Peng Guo, Wenjing Zhang, Weijiao Zhang, Jinghui Li, Huiying Guan, Weijia Zhang, Dongmei Luo, Jing Qi, and Hao Yue contributed to the acquisition and collation of data; Yue Huang, Yanan Zhang, Gaifen Liu, and Xiaomin Wang contributed to directing paper writing and data statistics; Wei Zhang contributed to the conception, design and implementation of the study and the supervision of paper writing.

## FUNDING INFORMATION

This study was supported by the National Key Research and Development Program of China (2016YFC1306300, 2016YFC1306000), the National Natural Science Foundation of China (81970992, 81571229, 81071015, 30770745, 82201639), the Capital's Funds for Health Improvement and Research (CFH) (2022‐2‐2048), the Key Technology R&D Program of Beijing Municipal Education Commission (kz201610025030), the Key Project of Natural Science Foundation of Beijing, China (4161004), the Natural Science Foundation of Beijing, China (7082032), the Project of Scientific and Technological Development of Traditional Chinese Medicine in Beijing (JJ2018‐48), the Capital Clinical Characteristic Application Research (Z121107001012161), the High Level Technical Personnel Training Project of Beijing Health System, China (2009‐3‐26), the Project of Beijing Institute for Brain Disorders (BIBD‐PXM2013_014226_07_000084), the Excellent Personnel Training Project of Beijing, China (20071D0300400076), the Project of Construction of Innovative Teams and Teacher Career Development for Universities and Colleges Under Beijing Municipality (IDHT20140514), the Beijing Healthcare Research Project, China (JING‐15‐2), the Basic‐Clinical Research Cooperation Funding of Capital Medical University, China (2015‐JL‐PT‐X04, 10JL49, 14JL15), the Natural Science Foundation of Capital Medical University, Beijing, China (PYZ2018077), and the Youth Research Funding, Beijing Tiantan Hospital, Capital Medical University, China (2015‐YQN‐14, 2015‐YQN‐15, 2015‐YQN‐17).

## CONFLICT OF INTEREST STATEMENT

The authors report no competing interests.

## CONSENT FOR PUBLICATION

Not applicable.

## Supporting information


Data S1.

## Data Availability

The data that support the findings of this study are available from the first author or the corresponding author upon reasonable request.
