# Supplementary material for: Role of nerve growth factor on cognitive impairment in patients with Alzheimer's disease carrying apolipoprotein E ε4
Source: CNS Neurosci Ther. 2023 Dec 19;30(6):e14560. doi: 10.1111/cns.14560 (PMC11163191; doi:10.1111/cns.14560)
Supplement: Supplementary file 1 — Data S1. [file CNS-30-e14560-s001.docx]

**Supplementary material**

**Assessment of cognitive function**

**Global cognitive function**

Global cognitive function of enrolled AD patients was assessed by Mini-Mental State Examination (MMSE)(Cockrell and Folstein, 1988) and Montreal Cognitive Assessment (MoCA)(Nasreddine et al., 2005).

MMSE is a 30-point test. First, orientation to time and place is evaluated. The immediate memory is evaluated by learning trial of three nouns and short-term memory recall is assessed after evaluating attention and calculation ability with a serial subtraction task. Language is assessed by repeating a sentence, following the instructions written on the card, performing a sentence, and saying a complete and meaningful sentence. Finally, visuospatial ability is assessed by copying an overlapping pentagon. Patients with illiteracy, primary education, or more than a junior education are identified as dementia when the MMSE score was below 17, 20 or 24 points, respectively. The lower the score of MMSE, the poorer the global cognitive function.

MoCA is a 30-point test administered in 10 minutes. The short-term memory recall task involves 2 learning trials of 5 nouns, and delayed recall is performed after approximately 5 minutes. Visuospatial ability is assessed using a clock-drawing task and a three-dimensional cube copy. Multiple aspects of executive function are assessed using an alternation task adapted from the Trail Making B task, a phonemic fluency task, and a two-item verbal abstraction task. Attention, concentration and working memory are evaluated using a sustained attention task (target detection using tapping), a serial subtraction task, and digits forward and backward. Language is assessed using a three-item confrontation naming task with low-familiarity animals (lion, camel and rhinoceros), repetition of two syntactically complex sentences, and the aforementioned fluency task. Finally, orientation to time and place is evaluated. The MoCA score ≤ 26 indicates cognitive impairment and 1 point is added if the educational level of an individual is less than 12 years. The lower the score of MoCA, the worse the global cognitive function.

**Individual cognitive domains**

**Memory**

Verbal memory was evaluated by Auditory Verbal Learning Test (AVLT), which consists of 5 learning trials in total(Guo et al., 2009). During each trial, a list of 12 novel words is read aloud with a 1-second pause between each word, and patients are asked to recall immediately as many words as possible. The above process is repeated 3 times, with scores named N1, N2, and N3, respectively. Patients are then asked to recall the 12 words again after 5-minute and 20-minute intervals, with scores named N4 and N5, respectively. Immediate recall, short-delayed recall and long-delayed recall are evaluated as AVLT N1-3, AVLT N4 and AVLT N5. Poor verbal delayed memory is indicated by the low score of AVLT.

Visual delayed memory was evaluated by Rey-Osterreithm Complex Figure Test (RCFT)-delayed recall(Shin et al., 2006). In the test, patients are asked to duplicate a complex figure within 10 minutes, with score named RCFT-imitation, and then drew the figure from memory after 25 minutes, with score named RCFT-delayed recall(Shin et al., 2006). Poor visual delayed memory is indicated by the low score of RCFT-delayed recall.

**Language**

Language was evaluated by Verbal Fluency Test (VFT) and Boston Naming Test (BNT). VFT consists of 3 parts(Mok et al., 2004). Patients firstly are asked to name as many animals as possible in a minute; then to list the names of as many household items as possible in a minute, and finally to alternate the names of animals and household items as many times as possible in a minute. There are no points given for items more than once, and there are points given for the number listed. The lower the score of VFT, the severer the language dysfunction.

BNT involves displaying the subjects with 30 pictures and then asking them to name each one(Katsumata et al., 2015). One point is given for each picture correctly answered, and the total number of correct answers is recorded. The lower the score of BNT, the severer the language dysfunction.

**Visuospatial ability**

In RCFT, patients are asked to duplicate a complex figure within 10 minutes, with score named RCFT-imitation. RCFT-imitation was used to evaluate visuospatial ability(Shin et al., 2006). A low score of RCFT-imitation suggests compromised visuospatial ability.

**Attention / Executive function**

Attention was evaluated by Trail Making Test (TMT)-A, Stroop Color-Word Test (SCWT)-A, SCWT-B and Symbol Digit Modalities Test (SDMT). The longer it takes to complete the test and the lower the score of the test, the poorer the attention of the individual patient. Executive function was evaluated by TMT-B and SCWT-C. The low scores of the two tests indicate impaired executive function.

TMT consists of TMT-A and TMT-B(Wei et al., 2018). TMT-A requires the subjects to draw a line between 25 consecutive numbers as quickly as possible without lifting their pencil. In TMT-B, except for number 1, all the other 24 numbers appear twice, which are square and circle shapes, respectively. Patients are asked to connect 25 consecutive numbers in sequence as quickly as possible without lifting their pencil, interconnecting the two shapes while connecting the numbers.

SCWT consists of 3 parts, including SCWT-A, SCWT-B and SCWT-C(Bondi et al., 2002). In SCWT-A, there are 50 words, including red, yellow, blue and green. Patients are asked to read each word correctly and quickly. In SCWT-B, there are 50 circles with four colors of red, yellow, blue and green. Patients are asked to read each color accurately and rapidly from left to right. In SCWT-C, there are 50 words of different colors that do not correspond to the color, and patients are asked to read the color of the words instead of the words itself.

The beginning of SDMT form are 9 unique geometric shapes paired with numbers ranging from 1 to 9. Below the key are rows of boxes with geometric shapes in the top boxes with rows of empty boxes. Patients are instructed to fill each empty box with the number that matches the shape using the key at the top of the page. To start, patients complete 10 practice items. Following the practice trial, patients are then instructed to fill as many boxes as possible in 90 seconds, matching numbers to the corresponding shape(Fellows and Schmitter-Edgecombe, 2019).

**Supplementary tables and figures**

**Supplementary Table 1 Association of *APOE* ε4 with cognitive function in AD patients**

|  | **Unadjusted** | | **Adjusted** | |
| --- | --- | --- | --- | --- |
|  | **β (95%CI)** | ***p*** | **β (95%CI)** | ***p*** |
| **Global cognitive function** |  |  |  |  |
| MMSE (points) | -2.29 (-4.84, 0.26) | 0.078 | -1.85 (-4.64, 0.93) | 0.190 |
| MoCA (points) | -2.75 (-4.96, -0.54) | **0.015*** | -2.31 (-4.58, -0.05) | **0.046*** |
| **Individual cognitive domain function** |  |  |  |  |
| Memory |  |  |  |  |
| AVLT N1-3 (points) | -2.51 (-4.45, -0.57) | **0.012*** | -2.35 (-4.51, -0.18) | **0.034*** |
| AVLT N4 (points) | -0.94 (-1.81, -0.06) | **0.036*** | -0.76 (-1.77, 0.25) | 0.138 |
| AVLT N5 (points) | -0.88 (-1.71, -0.06) | **0.037*** | -0.69 (-1.62, 0.24) | 0.145 |
| AVLT N1-5 (points) | -5.12 (-8.44, -1.79) | **0.003**** | -4.20 (-8.14, 0.25) | **0.037*** |
| AVLT N6 (points) | -1.10 (-1.95, -0.26) | **0.011*** | -1.01 (-1.94, -0.08) | **0.034*** |
| AVLT N7 (points) | -2.44 (-4.65, -0.24) | **0.030*** | -2.62 (-5.02, -0.21) | **0.033*** |
| RCFT-delayed (points) | -0.23 (-3.84, 3.37) | 0.898 | -0.29 (-4.09, 3.51) | 0.879 |
| Language |  |  |  |  |
| AFT (points) | -2.47 (-4.32, -0.62) | **0.009**** | -2.06 (-4.04, -0.09) | **0.040*** |
| VFT-H (points) | -2.07 (-3.81, -0.33) | **0.020*** | -1.49 (-3.44, 0.45) | 0.131 |
| VFT-alternating fluency (points) | -1.79 (-3.55, -0.03) | **0.046*** | -1.26 (-3.29, 0.76) | 0.220 |
| BNT (points) | -1.91 (-4.20, 0.37) | 0.100 | -2.10 (-4.47, 0.28) | 0.083 |
| Visuospatial ability |  |  |  |  |
| RCFT-imitation (points) | -1.41 (-6.66, 3.84) | 0.595 | -1.99 (-8.15, 4.17) | 0.523 |
| Attention / Executive function |  |  |  |  |
| TMT-A (points) | -0.38 (-3.02, 2.27) | 0.779 | -0.51 (-3.67, 2.65) | 0.750 |
| TMT-A (time) (minutes) | 0.47 (0.03, 0.90) | **0.036*** | 0.45 (0.21, 0.88) | **0.040*** |
| TMT-B (points) | -1.82 (-5.46, 1.82) | 0.323 | -2.18 (-6.20, 1.84) | 0.284 |
| TMT-B (time) (minutes) | 0.57 (0.01, 1.13) | **0.045*** | 0.34 (-0.29, 0.97) | 0.284 |
| SCWT-A (points) | -0.40 (-5.81, 5.01) | 0.884 | -0.86 (-7.00, 5.28) | 0.782 |
| SCWT-A (time) (minutes) | 0.08 (-0.20, 0.37) | 0.568 | 0.07 (-0.29, 0.43) | 0.707 |
| SCWT-B (points) | -2.86 (-8.08, 2.37) | 0.282 | -3.98 (-10.22, 2.25) | 0.208 |
| SCWT-B (time) (minutes) | 0.02 (-0.22, 0.27) | 0.852 | 0.01 (-0.28, 0.36) | 0.927 |
| SCWT-C (points) | -3.16 (-9.54, 3.22) | 0.329 | -4.07 (-11.75, 3.62) | 0.296 |
| SCWT-C (time) (minutes) | 0.06 (-0.34, 0.45) | 0.771 | -0.01 (-0.46, 0.43) | 0.952 |
| SDMT (points) | -3.54 (-9.68, 2.61) | 0.257 | -0.86 (-7.71, 6.00) | 0.805 |

Notes: Age, gender, age of onset, disease duration and education level were adjusted. **p* < 0.05. Abbreviation: *APOE, apolipoprotein E*; MMSE, Mini-Mental State Examination; MoCA, Montreal Cognitive Assessment; AVLT, Auditory Verbal Learning Test; AFT, Animal Fluency Test; VFT, Verbal Fluency Test; BNT, Boston Naming Test; RCFT, Rey-Osterrieth Complex Figure Test; TMT, Trail Making Test; SCWT, Stroop Color and Word Test; SDMT, Symbol Digit Modalities Test.


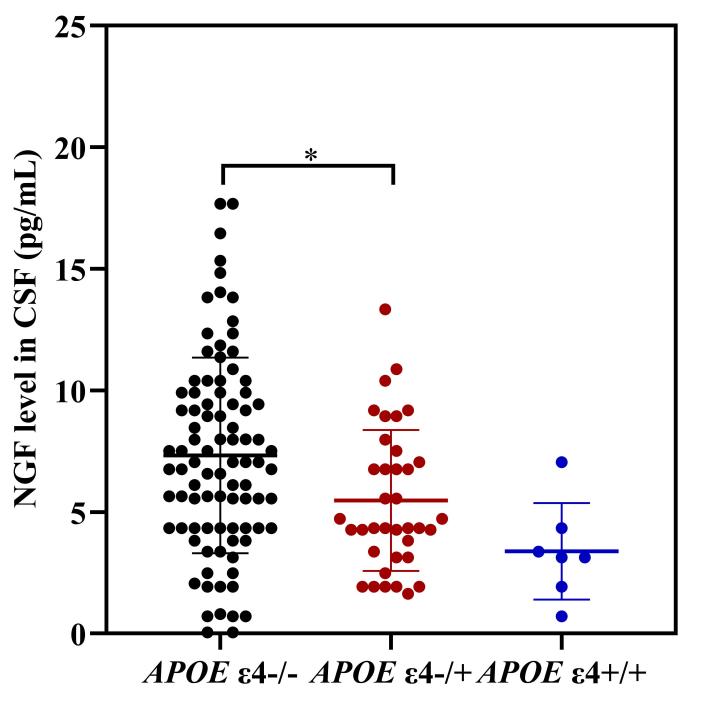


**Supplementary Figure 1 NGF level in CSF between the** ***APOE* ε4-/-, *APOE* ε4-/+, and *APOE* ε4+/+ groups.** *APOE* ε4-/-, *APOE* ε4 non-carrier; *APOE* ε4-/+, single *APOE* ε4 carriers; *APOE* ε4+/+, double *APOE* ε4 carriers. **p* < 0.05*: APOE* ε4-/- group vs *APOE* ε4-/+ group. Abbreviation: NGF, nerve growth factor; CSF, cerebrospinal fluid; *APOE, apolipoprotein E.*

**Supplementary Table 2 Association of the NGF level in CSF with cognitive functions in AD patients carrying *APOE* ε4**

|  | | **Unadjusted** | | | **Adjusted** | | |
| --- | --- | --- | --- | --- | --- | --- | --- |
|  | | **β (95%CI)** | ***p*** | | **β (95%CI)** | ***p*** | |
| **Global cognitive function** | |  |  | |  |  | |
| MMSE (points) | | 0.17 (-0.39, 0.74) | 0.540 | | 0.16 (-0.37, 0.68) | 0.532 | |
| MoCA (points) | | 0.24 (-0.31, 0.79) | 0.387 | | 0.27 (-0.22, 0.76) | 0.262 | |
| **Individual cognitive domain function** | |  |  | |  |  | |
| Memory | |  |  | |  |  | |
| AVLT N1-3 (points) | | 0.60 (0.11, 1.09) | **0.017*** | | 0.50 (0.11, 0.89) | **0.015*** | |
| AVLT N4 (points) | | 0.24 (0.10, 0.38) | **0.001**** | | 0.20 (0.05, 0.34) | **0.010*** | |
| AVLT N5 (points) | | 0.18 (0.04, 0.31) | **0.014*** | | 0.14 (-0.00, 0.29) | 0.054 | |
| AVLT N1-5 (points) | | 0.73 (0.11, 1.35) | **0.023*** | | 0.63 (0.03, 1.24) | **0.042*** | |
| AVLT N6 (points) | | 0.12 (-0.06, 0.29) | 0.176 | | 0.10 (-0.10, 0.30) | 0.306 | |
| AVLT N7 (points) | | 0.38 (-0.15, 0.91) | 0.149 | | 0.44 (-0.06, 0.94) | 0.079 | |
| RCFT-delayed (points) | | 1.93 (1.01, 2.84) | **＜0.001**** | | 1.29 (0.37, 2.22) | **0.010*** | |
| Language |  | | |  |  | |  |
| AFT (points) | | 0.18 (-0.29, 0.65) | 0.437 | | 0.35 (-0.10, 0.80) | 0.121 | |
| VFT-H (points) | | 0.15 (-0.28, 0.58) | 0.474 | | 0.32 (-0.02, 0.66) | 0.063 | |
| VFT (points) | | 0.33 (-0.04, 0.71) | 0.082 | | 0.41 (0.02, 0.79) | **0.041*** | |
| BNT (points) | | -0.13 (-0.77, 0.51) | 0.681 | | 0.34 (-0.25, 0.92) | 0.247 | |
| Visuospatial ability | |  |  | |  |  | |
| RCFT-imitation (points) | | 1.05 (-0.37, 2.48) | 0.143 | | -0.18 (-1.39, 1.76) | 0.810 | |
| Attention/Executive function | |  |  | |  |  | |
| TMT-A (points) | | 0.34 (-0.30, 0.98) | 0.288 | | 0.36 (-0.23, 0.95) | 0.220 | |
| TMT-A (time) (minutes) | | -1.03 (-0.22, 0.01) | 0.069 | | -0.90 (-0.20, 0.02) | 0.102 | |
| TMT-B (points) | | 0.75 (-0.13, 1.62) | 0.092 | | 0.69 (-0.17, 1.56) | 0.111 | |
| TMT-B (time) (minutes) | | -0.03 (-0.16, 1.00) | 0.638 | | -0.03 (-0.16, 0.15) | 0.970 | |
| SCWT-A (points) | | 0.26 (-1.23, 1.75) | 0.725 | | 0.19 (-1.22, 1.59) | 0.785 | |
| SCWT-A (time) (minutes) | | -0.10 (-0.08, 0.07) | 0.802 | | -0.03 (-0.12, 0.06) | 0.452 | |
| SCWT-B (points) | | -0.51 (-1.13, 2.15) | 0.534 | | 0.56 (-1.03, 2.14) | 0.474 | |
| SCWT-B (time) (minutes) | | -0.00 (-0.06, 0.05) | 0.888 | | -0.00 (-0.06, 0.06) | 0.943 | |
| SCWT-C (points) | | 0.88 (-0.85, 2.61) | 0.308 | | 0.91 (-0.74, 2.55) | 0.263 | |
| SCWT-C (time) (minutes) | | -0.03 (-0.14, 0.08) | 0.567 | | 0.01 (-0.11, 0.12) | 0.903 | |
| SDMT (points) | | 0.94 (-0.22, 2.10) | 0.108 | | 0.53 (-0.81, 1.86) | 0.413 | |

Notes: Age, gender, age of onset, disease duration, education level and BMI were adjusted. **p* < 0.05, ***p* < 0.01. Abbreviation: NGF, nerve growth factor; AD, Alzheimer’s disease; CSF, cerebrospinal fluid; *APOE, apolipoprotein E;* MMSE, Mini-Mental State Examination; MoCA, Montreal Cognitive Assessment; AVLT, Auditory Verbal Learning Test; AFT, Animal Fluency Test; Auditory Verbal Learning Test; VFT, Verbal Fluency Test; BNT, Boston Naming Test; RCFT, Rey-Osterrieth Complex Figure Test; TMT, Trail Making Test; SCWT, Stroop Color and Word Test; SDMT, Symbol Digit Modalities Test.

**References**

Bondi MW, Serody AB, Chan AS, Eberson-Shumate SC, Delis DC, Hansen LA, Salmon DP (2002) Cognitive and neuropathologic correlates of Stroop Color-Word Test performance in Alzheimer's disease. Neuropsychology 16:335-343.

Cockrell JR, Folstein MF (1988) Mini-Mental State Examination (MMSE). Psychopharmacology bulletin 24:689-692.

Fellows RP, Schmitter-Edgecombe M (2019) Symbol Digit Modalities Test: Regression-Based Normative Data and Clinical Utility. Archives of clinical neuropsychology : the official journal of the National Academy of Neuropsychologists 35:105-115.

Guo Q, Zhao Q, Chen M, Ding D, Hong Z (2009) A comparison study of mild cognitive impairment with 3 memory tests among Chinese individuals. Alzheimer disease and associated disorders 23:253-259.

Katsumata Y, Mathews M, Abner EL, Jicha GA, Caban-Holt A, Smith CD, Nelson PT, Kryscio RJ, Schmitt FA, Fardo DW (2015) Assessing the discriminant ability, reliability, and comparability of multiple short forms of the Boston Naming Test in an Alzheimer's disease center cohort. Dementia and geriatric cognitive disorders 39:215-227.

Mok EH, Lam LC, Chiu HF (2004) Category verbal fluency test performance in chinese elderly with Alzheimer's disease. Dementia and geriatric cognitive disorders 18:120-124.

Nasreddine ZS, Phillips NA, Bédirian V, Charbonneau S, Whitehead V, Collin I, Cummings JL, Chertkow H (2005) The Montreal Cognitive Assessment, MoCA: a brief screening tool for mild cognitive impairment. Journal of the American Geriatrics Society 53:695-699.

Shin MS, Park SY, Park SR, Seol SH, Kwon JS (2006) Clinical and empirical applications of the Rey-Osterrieth Complex Figure Test. Nature protocols 1:892-899.

Wei M, Shi J, Li T, Ni J, Zhang X, Li Y, Kang S, Ma F, Xie H, Qin B, Fan D, Zhang L, Wang Y, Tian J (2018) Diagnostic Accuracy of the Chinese Version of the Trail-Making Test for Screening Cognitive Impairment. Journal of the American Geriatrics Society 66:92-99.
